# Cumulative Lead Dose and Cognitive Function in Adults: A Review of Studies That Measured Both Blood Lead and Bone Lead

**DOI:** 10.1289/ehp.9786

**Published:** 2006-12-22

**Authors:** Regina A. Shih, Howard Hu, Marc G. Weisskopf, Brian S. Schwartz

**Affiliations:** 1 Division of Epidemiology, Statistics, and Prevention Research, National Institute of Child Health and Human Development, National Institutes of Health, Department of Health and Human Services, Rockville, Maryland, USA; 2 Department of Environmental Health, Harvard School of Public Health, Boston, Massachusetts, USA; 3 Channing Laboratory, Department of Medicine, Brigham and Women’s Hospital, Harvard Medical School, Boston, Massachusetts, USA; 4 Departments of Environmental Health Sciences and Epidemiology, Johns Hopkins Bloomberg School of Public Health and; 5 Department of Medicine, School of Medicine, Johns Hopkins University, Baltimore, Maryland, USA

**Keywords:** adults, blood, bone, cognitive function, lead, neurobehavior

## Abstract

**Objective:**

We review empirical evidence for the relations of recent and cumulative lead dose with cognitive function in adults.

**Data Sources:**

A systematic search of electronic databases resulted in 21 environmental and occupational studies from 1996 to 2006 that examined and compared associations of recent (in blood) and cumulative (in bone) lead doses with neurobehavioral outcomes.

**Data extraction:**

Data were abstracted after consideration of exclusion criteria and quality assessment, and then compiled into summary tables.

**Conclusions:**

At exposure levels encountered after environmental exposure, associations with bio-markers of cumulative dose (mainly lead in tibia) were stronger and more consistent than associations with blood lead levels. Similarly, in studies of former workers with past occupational lead exposure, associations were also stronger and more consistent with cumulative dose than with recent dose (in blood). In contrast, studies of currently exposed workers generally found associations that were more apparent with blood lead levels; we speculate that the acute effects of high, recent dose may mask the chronic effects of cumulative dose. There is moderate evidence for an association between psychiatric symptoms and lead dose but only at high levels of current occupational lead exposure or with cumulative dose in environmentally exposed adults.

In the development of the adult lead management guidelines (see Kosnett et al. 2007), a number of health outcomes adversely affected by lead exposure were discussed. Cognitive function was an important consideration of because of the growing number of studies in this area and increasing concern that cognitive function in adulthood may be affected by relatively low lead doses. In this article, we systematically review recent evidence concerning recent and cumulative lead dose and adult cognitive function.

## Measurement of lead dose

In reviewing studies of the health effects of lead, it is critical to understand the available lead biomarkers in terms of how they represent external exposure (in terms of timing, duration, magnitude, and accumulation); how they are influenced by metabolic factors (organ distribution, compartmental dynamics, the influence of physiologic factors); and how the combination of these considerations affects inferences regarding the health effects of lead (Hu et al. 2007). We conclude from these important methodologic issues that the most informative recent epidemiologic studies of lead’s impact on health are those that were able to derive estimates of both recent and cumulative lead exposure for each study participant. To achieve this end with the greatest precision and accuracy, such studies have incorporated measurements of lead in both blood (whole blood, using standard chemical assays such as graphite furnace atomic absorption spectroscopy) and bone [using noninvasive *in vivo* K-shell X-ray fluorescence (KXRF) instruments].

Blood lead levels measured in epidemiologic studies with valid instruments and standardized calibration and quality control procedures have been reported in the literature for > 35 years. Bone lead levels measured by *in vivo* KXRF were begun in some research laboratories in the 1980s, but it was not until the mid-1990s that reports began to emerge of KXRF-measured bone lead levels in relation to potential health indicators from epidemiologic studies with sufficient sample sizes (for example, ≥ 100 subjects) to have substantial statistical power. Thus, in this review we summarize all studies to date that measure cognitive function and both blood and bone lead levels (or acceptable surrogate for cumulative lead dose).

## Published reviews of relevance to this review

We begin our review with a discussion of three other reviews on the topic of lead dose and cognitive function (Balbus-Kornfeld et al. 1995; Goodman et al. 2002; Meyer-Baron and Seeber 2000). Balbus-Kornfeld et al. (1995) reviewed the evidence on cumulative lead exposure and cognitive function from studies published from 1976 to 1991. Among 21 unique studies that were identified at the time of the authors’ review, none used a biomarker of cumulative dose. Of the four longitudinal studies, all were small (mean sample size in the analysis of 47 lead-exposed subjects), with relatively low follow-up rates and relatively short durations of follow-up. The authors thus concluded that the available literature provided inadequate evidence to conclude whether cumulative exposure or absorption of lead adversely affected cognitive function in adults.

Goodman et al. (2002) and Meyer-Baron and Seeber (2000) are reviewed here because they had generally opposite conclusions, which led to considerable controversy and discussion (Goodman et al. 2001; Schwartz et al. 2002; Seeber and Meyer-Baron 2003; Seeber et al. 2002). The Goodman et al. (2002) article was funded by the German Battery Association, apparently in anticipation of consideration in Germany of lowering the blood lead standard in lead workers (Seeber and Meyer-Baron 2003). Goodman et al. (2002) reviewed 22 studies published between 1974 and 1999 with the expressed aim of evaluating associations between moderate blood lead levels and neurobehavioral test scores after occupational exposure to lead. Studies were included if the central tendency for blood lead levels was < 70 μg/dL, the numbers of exposed and unexposed were reported, and test score arithmetic means and measures of variability were reported for exposed and unexposed workers (Goodman et al. 2002). The authors concluded that none of the individual studies were conclusive or adequate in providing information on the effects of lead on cognitive function and called for prospective studies that would evaluate cognitive function before and after exposure. There was no discussion about whether examining relations of blood lead levels with cognitive function was the most relevant question if the hypothesis was that cumulative lead dose was most important to cognitive function. There was little explicit discussion of whether lead may have acute effects as a function of recent dose, and chronic effects as a function of cumulative dose, or how this could be assessed by review of epidemiologic studies.

Meyer-Baron and Seeber (2000) performed a meta-analysis of 12 studies using selection criteria similar to Goodman et al. (2002) but also with the requirement for reporting means and standard deviations of dependent variables (Meyer-Baron and Seeber 2000). They concluded that there were obvious neurobehavioral deficits at current blood lead levels < 40 μg/dL. Again, the focus was on associations with blood lead levels, and there was little formal discussion about which lead biomarker was most relevant to hypotheses about how cumulative lead dose may influence cognitive function. Thus, this is the first review to evaluate epidemiologic studies that distinguish between the acute effects of recent dose from the chronic effects of cumulative dose.

## Methods

### Methodologic considerations for relations of lead dose and cognitive function

Many methodologic issues of relevance to the epidemiologic investigation of lead and cognitive function have been addressed elsewhere in this minimonograph (Hu et al. 2007). When evaluating the associations of cumulative lead dose with cognitive function, it is important to acknowledge that nonoccupational sources of lead exposure were present for all members of the general population, including lead workers throughout the early part of this century until public health interventions progressively removed lead from gasoline and many consumer products during the 1970s and 1980s (Agency for Toxic Substances and Disease Registry 1999; Annest et al. 1983; Pirkle et al. 1998). Lead remains a low-level and ubiquitous neurotoxicant in the environment and is found in measurable levels in all individuals (Hoppin et al. 1995). Thus, current tibia lead levels represent a mix of occupational and environmental exposures. This review does not try to determine whether the main source of lead was occupational or environmental but rather focuses on whether lead in blood or bone is associated with adverse cognitive outcomes in adults.

### Identification of studies

We conducted a systematic literature review of the association between blood and bone lead biomarkers and cognitive functioning in adults. Our aim was to select studies that compared markers of both recent and cumulative lead dose in their relations with cognitive function. Both occupationally and environmentally exposed adult populations were included. We searched the PubMed (National Library of Medicine 2006) and PsycINFO databases (American Psychological Association 2006) for epidemiologic studies using keywords such as blood, bone, lead, cumulative, cognitive, and neurobehavior. There were no date or language restrictions. From the identified publications and relevant review articles, we examined reference lists to locate additional studies that measured both recent and cumulative lead dose. This includes blood lead levels, bone lead levels, or a surrogate measure of cumulative lead dose such as integrated blood lead (IBL), area under the curve of blood lead levels over time, or the product of blood lead level and employment time. Studies were not considered for the review if they *a*) contained no original research, *b*) were conducted on nonhuman subjects, *c*) were case reports, *d*) contained no standardized neurocognitive assessment outcomes, or *e*) lacked measures of both recent and cumulative lead dose.

### Data abstraction

We abstracted data from articles meeting the selection criteria. Study quality was assessed with the following criteria: *a*) exposure was assessed at an individual level; *b*) exposure was assessed with a biomarker; *c*) cognitive outcomes were objective, standardized tests; *d*) statistical adjustment for potential confounders including age, sex (in studies with both men and women), and education; *e*) data collection was similar in exposed and nonexposed participants; *f* ) time period of study was the same in exposed and nonexposed participants; and *g*) there was a detailed description of the approach to data analysis. We decided not to try to derive a pooled estimate across studies of the associations of lead dose biomarkers with cognitive function because of differences in methods for subject selection, blood and bone lead measurements, neurobehavioral outcomes, approach to regression modeling, and presentation of results across studies. Pooled estimates from metaanalysis also can be highly influenced by decisions regarding how and whether to pool certain results. We thus decided to present details for each study and discuss them in turn.

## Results

### Overview of evidence

We identified three main types of studies that reported cross-sectional or longitudinal associations of blood and bone lead levels with cognitive function. These were of *a*) environmentally exposed individuals in the general population, *b*) workers with current occupational exposure, and *c*) former lead workers without current occupational exposure to lead. We have summarized these studies in Table 1, provided details in Table 2, and discuss them in order below.

### Studies of adults without occupational lead exposure

We identified six articles from three studies [i.e., residents near a lead smelter, the Normative Aging Study (NAS), and the Baltimore Memory Study] that evaluated subjects with mainly environmental exposure to lead (Tables 1 and 2). One study of young adults 19–29 years of age compared 257 individuals with high childhood blood lead levels from exposure 20 years previously from a lead smelter to 276 age- and sex-matched controls. This study found impairment on many cognitive tests among the highly exposed group, but minimal association on most tests with tibia lead levels measured during young adulthood (Stokes et al. 1998).

Four articles from the NAS reported associations of blood and bone lead levels in a cohort of older men. One of these articles (Payton et al. 1998) was a first report that examined scores on a large battery of cognitive tests of a small sample (*n* = 141) of NAS participants. This was subsequently followed up with a report on a much larger number of NAS participants (*n* = 1,089 with blood lead levels and *n* = 760 with bone lead levels, 412–515 of whom took different tests twice approximately 3.5 years apart) (Weisskopf et al. 2007*)*. Cross-sectional analyses in the original report found that increased blood lead levels across a relatively low range of levels [mean ± SD = 5.5 ± 3.5 μg/dL) were a stronger predictor, compared with tibia or patella lead levels, of poorer performance on tests of speed, verbal memory, vocabulary, and spatial copying skills. However, this was not confirmed in the larger, cross-sectional analysis, except possibly for scores on a vocabulary test (Weisskopf et al. 2007). Conversely, in longitudinal analyses, the larger study found more decline over time on almost all cognitive tests associated with both higher patella and higher tibia bone lead levels, with the associations reaching statistical significance for pattern comparison and spatial copying skills. An earlier, similar longitudinal analysis by Weisskopf et al. (2004) in this same population reported that patella lead levels were significantly associated with a decline in Mini-Mental State Examination (MMSE; Folstein et al. 1975) score over time. A slightly smaller association was observed with tibia lead levels, whereas no association was observed with blood lead levels. In cross-sectional analyses of the same population, higher blood lead levels were a stronger predictor of poorer performance on the MMSE, as were higher patella and tibia bone lead levels (Payton et al. 1998; Wright et al. 2003).

In a study of almost 1,000 persons 50–70 years of age randomly selected from the general population in the Baltimore Memory Study (BMS), a cross-sectional analysis showed that relatively low current blood lead levels were not associated with cognitive domain scores. However, moderate tibia lead levels (mean ~ 19 μg/g) were significantly associated with worse performance in all seven cognitive domains (Shih et al. 2006). Thus, in the environmental studies of older adults, the most consistent findings across studies are associations between bone lead levels and cognitive function. The associations in the BMS were cross-sectional, whereas the predominant associations in the NAS were with change in cognitive function over time, although a significant cross-sectional association with MMSE score was also observed in this sample. Taken together, these data suggest that at environmental exposure levels, the effects of cumulative exposure are more pronounced than recent effects of current exposure. The absence of associations in the Stokes et al. (1998) study could be because of the younger age of studied subjects, the very low current blood and tibia lead levels, or the inadequacy of tibia lead in the third decade of life to estimate early life dose (Hoppin et al. 2000).

### Studies of occupationally exposed workers

Fifteen articles were identified of workers with current or past occupational exposure to lead. Eight of these studies used a surrogate measure of cumulative lead dose (i.e., IBL) rather than a direct measure of lead in bone. Among these studies, which compared blood and IBL lead dose, when the lead exposure was primarily current (e.g., relatively high blood lead levels), most studies found an association between increasing blood lead values and worse cognitive function (Barth et al. 2002; Bleecker et al. 1997; Lucchini et al. 2000). However, studies in which the exposure was primarily in the past demonstrated that surrogate measures of cumulative dose were a stronger predictor of worse cognitive function compared with blood lead levels (Bleecker et al. 2005; Chia et al. 1997; Lindgren et al. 1996). Studies that used bone lead levels as a direct indicator of retained cumulative lead dose are summarized below.

One study of currently exposed lead workers in South Korea (*n* = 803) found strong and consistent associations of blood lead levels with worse cognitive function after adjustment for covariates, but tibia lead levels were not as consistently associated (Schwartz et al. 2001). The same null findings for bone lead levels were observed in two smaller studies, one with male smelter workers (*n* = 57) in whom finger bone (mixed trabecular and cortical tissue) lead levels were measured (Osterberg et al. 1997). The second article describes the study of a sample of 54 storage battery workers in whom tibia and calcaneus lead levels were measured (Hanninen et al. 1998). This is the only study published to date to report an association between IBL and cognitive outcomes in which there was a lack of an association with bone lead levels. Both these studies used early XRF techniques (e.g., KXRF with cobalt-57) with higher limits of detection that have not been commonly used since, and this use makes the findings more difficult to interpret. Bleecker et al. (1997), in a study similar to the one by Schwartz et al. (2001), reported stronger and more consistent associations of blood lead measures and neurobehavioral test performance compared to tibia lead levels.

In the South Korean lead workers with current occupational exposure, a longitudinal analysis was performed to separate recent lead dose (measured as blood lead levels) from cumulative lead dose (measured as tibia lead levels), and acute effects from chronic effects in 575 subjects with complete data across the three study visits (Schwartz et al. 2005). The authors reported significant cross-sectional associations of blood lead levels with lower executive ability and manual dexterity test scores, with some evidence also for a longitudinal association of changes in blood lead levels with neurobehavioral decline. Tibia lead levels were more consistently associated with longitudinal declines in manual dexterity, executive abilities, neuropsychiatric symptoms, and peripheral sensory functioning than change in blood lead levels. The authors concluded that lead was associated with worse cognitive function in two ways: an acute effect of recent dose and a chronic effect of cumulative dose. The authors also discussed that contrasting associations with blood and tibia lead levels could be due to the following: *a*) tibia and blood lead levels are biologically related and blood lead is in equilibrium with bone lead stores; *b*) the error in measurement of tibia lead levels is larger than that for blood lead; *c*) controlling for cross-sectional associations could obscure longitudinal ones; and *d*) lead in blood reflects recent external exposure, and is in equilibrium with bone lead stores, possibly taking away explained variance from bone lead associations via this correlation in cross-sectional analyses.

Results of a cross-sectional analysis of former organolead workers showed that higher peak tibia lead levels (range, –2.2 to 105.9 μg/g) were related to poorer functioning on a number of cognitive tests, including those assessing manual dexterity, executive ability, verbal intelligence, and verbal memory (Stewart et al. 1999). In a longitudinal analysis in this same population, among 535 lead workers exposed a mean of 16 years before, increases in peak tibia lead levels [mean ± SD = 22.6 ± 16.5 μg/g] but not in blood lead levels predicted declines over time in these same domains in addition to visual memory (Schwartz et al. 2000). This finding indicates that even many years after high lead exposure, and in the absence of high current lead exposure, cumulative lead dose may exert progressive effects on cognitive functioning (Links et al. 2001).

### Lead exposure and psychiatric symptoms

Several lines of evidence suggest that increased blood lead levels are associated with psychiatric symptoms in adults, such as depression, anxiety, irritability, and anger. For example, a cross-sectional analysis of 107 occupationally exposed individuals showed increased rates of depression, confusion, anger, fatigue, and tension as measured by the Profile of Mood States (POMS; McNair et al. 1971) among those with blood levels > 40 μg/dL (Baker et al. 1983). Maizlish et al. (1995) found that current and cumulative measures of blood lead levels in currently exposed lead workers were associated with tension, anxiety, hostility, and depression measured by the POMS questionnaire. Lindgren et al. (1996) examined the POMS’ factor structure in retired lead smelter workers and showed that the resulting “general distress” factor was significantly related to IBL but not to current blood lead level.

In occupationally exposed South Korean lead workers, tibia lead levels were significantly associated with more depressive symptoms measured by the Center for Epidemiologic Studies Depression scale (CES-D; Radloff 1977) after adjusting for age, sex, education, job duration, and blood lead level (Schwartz et al. 2001). However, only one recent study has examined a direct measure of cumulative dose with bone measurements in a community sample (Rhodes et al. 2003). These authors used the Brief Symptom Inventory (BSI; Derogatis and Melisaratos 1983) to show that patella bone lead levels were associated with an increased risk of anxiety and depression sub-scale scores. The logistic regression estimate for the phobic anxiety subscale was statistically significant (*p* < 0.05), as well as for the combined measure of all three BSI subscales (anxiety, depression, and phobic anxiety).

Psychiatric symptoms, specifically symptoms of depression, potentially share the same neural substrates with components of cognition, and thus may be important to late-life cognitive functioning. Compared with nondepressed elderly individuals, depressed elderly perform more poorly on tests involving attention, memory encoding, and retrieval. However, intelligence tests are more resistant to these effects of depression (Arnett et al. 1999; Naismith et al. 2003; Weingartner et al. 1981). Depressive symptoms (as measured by the CES-D) are positively associated with both the risk of Alzheimer disease and a steeper rate of cognitive decline (Wilson et al. 2002). Because late-life symptoms of depression are closely associated with dementia, investigators have put forth a number of hypotheses that suggest depression *a*) may be a risk factor for cognitive decline, *b*) has risk factors in common with dementia, *c*) is an early reaction to declining cognition, and *d*) influences the threshold at which dementia emerges [for review see Jorm (2000)]. The exact temporal and mechanistic relation remains unclear. Regardless of the exact relation between depressive symptoms and cognitive function, however, the assessment of the impact of lead exposure on these outcomes is not compromised. Whatever the associations with these outcomes, they would still be attributed to lead—that is, even if depressive symptoms lead to worse cognitive performance, and lead leads to symptoms of depression, the cognitive impairment as a result of that depression could still be considered part of the total effect of lead.

### Lead–gene interactions

In the former organolead worker studies discussed above, possessing at least one apolipoprotein E (*APOE)* ɛ *4* allele magnified the negative cross-sectional association of tibia lead levels with performance on the cognitive domains of executive ability, manual dexterity, and psychomotor skills (Stewart et al. 2002). No direct effects of the *APOE* ɛ *4* allele were observed on cognitive function in this study, presumably because of the sample’s younger age (range, 41–73 years). Other studies have found that *APOE* ɛ *4* modifies dementia outcome in individuals with previous traumatic head injury, suggesting that *APOE* ɛ *4* plays a role in recovery from brain insults (Mayeux et al. 1995), which may be extended to include insult from lead exposure.

## Discussion

### Summary of evidence for a causal relationship

The literature on associations of recent and cumulative dose biomarkers with cognitive function has grown impressively since the 1995 review (Balbus-Kornfeld et al. 1995). We believe sufficient evidence exists to conclude that there is an association between lead dose and decrements in cognitive function in adults. Overall, while the association between blood lead levels and cognitive function is more pronounced in occupational groups with high current lead exposures, associations between bone lead levels and cognitive function are more evident in studies of older subjects with lower current blood lead levels, particularly in longitudinal studies of cognitive decline.

#### Consistency of associations

Following is a summary of the findings from each of the three types of populations. First, cross-sectional studies of currently exposed lead workers showed that associations of blood lead levels and cognitive function were clearer than the associations for tibia, patella, or calcaneus lead levels, perhaps because the acute effects of recent dose in an occupational setting masked the chronic effects of cumulative lead dose. Second, previously exposed occupational populations demonstrated a stronger association between cumulative lead dose measured in tibia bone with cognitive deficits compared with blood lead levels. The two studies that deviated from these otherwise consistent findings may not have had sufficient power to detect any associations (*n* < 60). Last, studies of environmentally exposed adults who had notably higher exposures in the past suggest that bone lead level is more consistently associated with performance on cognitive tests than is blood lead level. The domains associated with lead dose do not differ in general by lead biomarker (blood, tibia, patella). The cognitive domains consistently associated with each biomarker in both environmental and occupational studies on adults include verbal and visual memory, visuospatial ability, motor and psychomotor speed, manual dexterity, attention, executive functioning, and peripheral motor strength. Comparisons of lead and psychiatric symptom associations in previously and currently exposed samples lend credence, although perhaps at higher thresholds than for cognitive outcomes, that neurobehavioral functioning is consistently associated with blood lead when exposure is currently high (e.g., occupational) and bone lead when exposure is primarily from past chronic exposure.

These associations exist in multiple settings, including both occupational and non-occupational, in men and women, and in populations with diversity by socioeconomic status and race/ethnicity. This reduces the likelihood of associations by statistical chance or due to unmeasured confounding. However, this consistency cannot completely rule out the possibility of uncontrolled confounding or effect modification (Martin et al. 2006; Shih et al. 2006). In addition, in studies of general populations with diversity by socioeconomic status and race/ethnicity, the ability to disentangle social, cultural, and biological factors from the “independent” influence of lead dose may be a futile exercise (Weiss and Bellinger 2006).

#### Strength of association

The strength of associations between lead and cognitive function is strong and can be compared to the influence of age on cognitive function. The comparative magnitude of these effects has been reported in several studies. In currently exposed lead workers, cross-sectional associations showed that a 5-μg/dL increase in blood lead was equivalent to an increase of 1.05 years in age (Schwartz et al. 2001). The magnitude of cross-sectional associations with tibia lead levels in the BMS was moderate to large. A proportion comparison of the direct effect of age and the direct effect of tibia lead levels on cognitive outcomes demonstrated that the magnitude of the association with tibia lead levels was moderate to large, equivalent to 22–60% of the magnitude of the age effect in its relations with cognitive domain scores. Specifically, an interquartile range increase in tibia lead levels was equivalent to 2–6 more years of age at baseline across all seven domains (Shih et al. 2006).

Longitudinal analyses in the NAS observed that an interquartile range higher patella lead level was approximately equivalent to that of aging 5 years in relation to the baseline MMSE score (Weisskopf et al. 2004) and an interquartile range higher bone (patella or tibia, depending on the specific cognitive outcome) lead level was approximately equivalent to that of aging 1 year in relation to the baseline test scores on a battery of cognitive tests (Weisskopf et al. 2007).

#### Specificity

Lead has adverse effects on many other health outcomes in addition to cognitive function. This is not surprising given lead’s numerous biologic effects, including calcium agnonism and antagonism (Ferguson et al. 2000), binding to sulfhydryl and carboxyl groups on proteins, and activation of nuclear transcription factors (Ramesh et al. 2001), for example. It is thus not surprising that lead’s toxicity is not specific to the brain and we do not believe this lack of target organ specificity diminishes the inference for a causal relationship between lead and cognitive dysfunction.

#### Temporal relationship

Associations between lead biomarkers and cognitive outcomes have been demonstrated in both cross-sectional and longitudinal studies. In several of the longitudinal studies, change in cognitive function was explicitly modeled in relation to preceding lead dose or in relation to change in lead dose. In either case, the temporality condition is met. In addition, as bone lead is a measure that ascertains prior dose, even in cross-sectional analyses, analysis of bone lead with cognitive test scores evaluates lead dose that preceded current cognitive performance; thus, while cognitive assessment is cross-sectional, dose assessment is retrospective and cumulative. This again would minimize concerns about incorrect temporal relations.

#### Biological gradient (dose–effect relations)

Nearly all reviewed studies found a dose–effect relation for blood lead, bone lead, or both. Existing studies do not allow determination of a threshold dose for either blood lead or bone lead or the shape of the dose–effect relationship at low dose levels. Associations have been observed in populations with mean blood lead levels as low as 4.5 μg/dL (Wright et al. 2003) and mean tibia lead levels as low as 18.7 μg/g (Shih et al. 2006).

#### Biologic plausibility and experimental data

Lead adversely affects the brain in a variety of ways. Lead is thought to increase oxidative stress, induce neural apoptosis, influence neurotransmitter storage and release, and damage mitochondria. The ability of lead to substitute for calcium allows it to affect calcium-mediated processes and pass through the blood–brain barrier. It may also interfere with zinc-dependent transcription factors, altering the regulation of genetic transcription (Zawia et al. 2000). Animal studies indicate that the accumulation of lead in the brain is generally uniform (Widzowski and Cory-Slechta 1994), although the hippocampus and limbic system, prefrontal cerebral cortex, and cerebellum are clearly principal sites of the effects of lead (Finkelstein et al. 1998). Low lead levels in rats produce structural changes in the hippocampus (Cory-Slechta 1995), a brain region critical for learning and memory (Eichenbaum 2001), which is consistent with the finding of learning and memory deficits in lead-exposed individuals.

Blood lead level is a measure of current biologically active lead burden and is therefore a better marker of the acute effects of recent lead dose. These are likely to be effects on neurotransmission and calcium enzyme-dependent processes such as synaptic plasticity. This could lead to circulating blood lead impairing, for example, information storage and retrieval mechanisms or processing speed, which have been suggested to impair performance on cognitive tests (Salthouse 1996a, 1996b). Lead levels in bone are a measure of cumulative dose over decades as well as a source of lead in the body that is available for mobilization into blood, especially during periods of increased bone turnover (e.g., pregnancy, puberty). Although lead stored in bone is not directly harmful to the brain, the cumulative effects of chronic lead exposure are likely to be related to oxidative stress and neuronal death and could impair cognitive function, for example, by reducing the capacity of specific regions to process information, or by impairing diffuse ascending projection systems such as the midbrain cholinergic and dopaminergic cells.

Lead may also influence cognitive function indirectly through its effects on blood pressure, hypertension, or homocysteine levels. Increased homocysteine levels, a well-known risk factor for cardiovascular disease, have also been associated with risk for poorer cognitive functioning (Dufouil et al. 2003; Schafer et al. 2005a) and risk for dementia (Hogervorst et al. 2002; McCaddon et al. 2003; Selley 2003). Homocysteine is neurotoxic to the central nervous system by influencing neurotransmitter synthesis, and causing excitotoxicity and cell death (McCaddon and Kelly 1992; Parnetti et al. 1997). Blood lead levels were associated with homocysteine levels as well, although the direction of causality has yet to be determined (Guallar et al. 2006; Schafer et al. 2005b). Both blood and bone lead levels have been linked with blood pressure and hypertension in community-based samples of older adults (Martin et al. 2006; Nash et al. 2003) and occupationally exposed populations (Glenn et al. 2003, 2006). Hypertension has also been identified as a potential risk factor for dementia (Birkenhager and Staessen 2006; Hayden et al. 2006; Skoog and Gustafson 2006). Thus, lead may indirectly play a role in cognitive declines by way of poor vascular health.

We believe the effect modification by *APOE* genotype offers strong biologic plausibility to the inference that lead causes cognitive dysfunction (Stewart et al. 2002). The *APOE* ɛ *4* allele is a risk factor for lateonset Alzheimier disease (Corder et al. 1993; Meyer et al. 1998; Saunders et al. 1993), hippocampal atrophy (Moffat et al. 2000), and senile plaques (Zubenko et al. 1994). It appears that the *APOE* ɛ *4* allele lowers the age of onset of the disease and accelerates age-related cognitive decline (Meyer et al. 1998). Mechanistically, *APOE* ɛ *4* is involved in the recovery response of injured nerve tissue (Poirier and Sevigny 1998), with the *APOE* ɛ *4* allele having reduced ability to promote growth and reduced antioxidant properties (Miyata and Smith 1996; Teter et al. 1999; Yankner 1996). The interaction of *APOE* genotype with tibia lead level may be related to an impaired ability to counteract injury from lead exposure among *APOE* ɛ *4* carriers.

Another recent study also offers biologic plausibility. In the former organolead workers, tibia lead level was associated with the prevalence and severity of white matter lesions on brain MRI, using the Cardiovascular Health Study white matter grading system (Stewart et al. 2006). Tibia lead level was also associated with smaller volumes on several regions of interest ranging from large (e.g., total brain volume, lobar gray and white matter volumes) to small (e.g., cingulate gyrus, insula, corpus callosum). As volume can decline because of changes in cell number, synaptic number or density, or other changes in cellular architecture, these findings reinforce evidence that lead may cause a persistent change in the brain that is associated with progressive declines in cognitive function.

### Public health implications

The removal of lead from gasoline, paint, and most other commercial products has succeeded in dramatically reducing environmental sources of lead exposure, and this has been reflected by the parallel declines in mean blood lead levels in Americans over the same time frame. However, lead has accumulated in the bones of older individuals, and especially those of lead workers exposed at the continued higher levels encountered in lead-using workplaces. Thus, past use of lead will continue to cause adverse health effects even when current exposures to lead are much lower than in the past. Lead in bone is not directly harmful to the central nervous system, and most of the structural and neurochemical damage is likely to have occurred decades ago. Nevertheless, lead in bone might serve as a source from which lead can be mobilized into blood, and potentially cross the blood–brain barrier. The chronic effects of lead may account for a proportion of cognitive aging; future research will be able to determine whether the chronic effects of cumulative lead dose alter the trajectory of normal cognitive aging. Research efforts should be directed to development of preventive interventions for both lead-associated cognitive decline with aging from past exposures, as well as the mobilization of current bone lead stores into the circulatory system leading to new health effects.

Cognitive aging occurs in conjunction with the normal biological aging process. It remains to be determined whether lead affects cognitive aging in adults by permanently reducing brain circuitry capacity thereby lowering baseline cognitive functioning, or by inducing steeper declines in cognitive functioning, leading to abnormal cognitive aging. It may be that lead influences cognitive health through its relationship with depressive symptoms, hypertension, or homocysteine levels, all of which influence cognitive impairment and risk of dementia. Future investigations should explicitly account for these complex causal pathways, and also determine whether chronic effects of cumulative lead dose increases the risk for such clinically relevant syndromes as mild cognitive impairment (Petersen et al. 1999).

## Figures and Tables

**Table 1:** Study characteristics of 21 articles on adult cognitive function (1996–2006) with biomarker measures of recent and cumulative lead dose.

Article feature	No. (percent of total no. of papers)
Main source of lead exposure	
Occupational	15 (71.4)
Environmental	6 (28.6)
Demographics	
Age (years)	
Mean < 50	15 (71.4)
Mean ≥ 50	6 (28.6)
Sex	
> 80% male	16 (76.2)
≤ 80% male	5 (23.8)
Race/ethnicity	
Mixed	1 (4.8)
Not mixed (> 80% one group)	14 (66.6)
Not reported	6 (28.6)
Type of lead dose^a^	
Blood lead (μg/dL)	
Peak/median/mean < 10	10 (47.6)
Peak/median/mean ≥ 10	11 (52.4)
Tibia lead (μg/g)	
Mean < 25	9 (42.9)
Mean ≥ 25	5 (28.6)
Patella lead (μg/g)	
Mean < 25	1 (4.8)
Mean ≥ 25	4 (19.0)
Cumulative dose measure	
Tibia	13 (61.9)
Patella	5 (28.6)
Integrated blood lead	8 (38.1)
Other	3 (14.3)

a Time-integrated blood lead was not summarized here because of differences in the way it is calculated for each study.

**Table 2:** Detailed summary and main findings of studies on cognitive function with recent and cumulative lead dose biomarkers.

Author	Sample size (no.)	Design	Percent male [mean age in years (SD)]	Race/ethnicity (%)	Source of Pb exposure	Primarily current/past exposure	Lead dose measure [mean (SD)]	Covariates adjusted for outcome measures	Summary of findings
Nonoccupational lead exposure									
Stokes et al. 1998	257 (E) 276 (R)	XS	47.7% (E) 24.3 (3.2) 49.6% (R) 24.2 (3.0)	White (E) 98 White (R) 94.2	Resided near lead smelter during childhood (E) Random sample of licensed drivers (R)	Past (E) ~ 20 years prior	Blood: (E) 2.9 (3.3) (R) 1.6 (1.4) Tibia: (E) 4.6 (range, –28.9 to 37) (R) 0.6 (range, –46.4 to 17.4)	Age, education, sex, height, BMI Battery of tests—6 domains	Dichotomized exposure group associated with neurobehavioral outcomes, but no significant associations between tibia lead and neurobehavioral outcomes
Payton et al. 1998	141	XS	100% 66.8 (6.8)	White 94	Environmental (Normative Aging Study)	Past	Blood: 5.5 (3.5) Tibia: 22.5 (12.2) Patella: 31.7 (19.2)	Age, education Battery of tests— 8 domains	Blood lead significant predictor of performance on speed, memory, spatial copying, and vocabulary Tibia lead associated with pattern memory and spatial copying Patella lead had less significant relationships with test scores than tibia lead
Wright et al. 2003	736 blood, tibia, patella lead 295 blood only	XS	100% 68.2 (6.9)	White 94	Environmental (Normative Aging Study)	Past	Blood: 4.5 (2.5) Tibia: 22.4 (15.3) Patella: 29.5 (21.2)	Age, education, alcohol intake MMSE score < 24	Blood lead OR = 1.21 (95% CI, 1.07–1.36) for MMSE < 24 Tibia lead OR = 1.02 (95% CI, 1.00–1.03) for MMSE < 24 Patella lead OR = 1.02 (95% CI, 1.00–1.04) for MMSE < 24 Patella and blood lead levels modified the effect of increasing age on MMSE score
Weisskopf et al. 2004	466 F/U = 61.9%	L	100% 67.4 (6.6)	White 94	Environmental (Normative Aging Study)	Past	Median [IQR] Blood: 4 [3, 5] Tibia: 19 [12, 26] Patella: 23 [15, 35]	Age, smoking, education, alcohol intake, and years between MMSE tests Change in MMSE score	Null association between baseline blood lead and change in MMSE Patella lead significantly associated with decline in MMSE (an IQR higher patella lead = ~ 5 years of aging on baseline MMSE) Tibia lead similar to patella but not quite significant
Weisskopf et al. 2007	1,089 blood 761 tibia 760 patella	XS and L	100% 68.7 (7.4)	White 98	Environmental (Normative Aging Study)	Past	Median [IQR] Blood: 5 [3, 6] Tibia: 20 [13, 28] Patella: 25 [17, 37]	Age, age squared, education, smoking, and alcohol intake Battery of 10 cognitive tests	XS analysis: blood lead significant predictor of performance on vocabulary test L analysis: tibia lead associated with pattern comparison L analysis: patella lead associated with pattern comparison and spatial copying
Shih, et al. 2006	994	XS	34.1% 59.4 (6.0)	African American 40.1	Environmental (Baltimore Memory Study)	Past	Blood: 3.5 (2.2) Tibia: 18.7 (11.2)	Series of 5 models adjusting for age, sex, *APOE e4* allele, education, race, wealth Scores in 7 cognitive domains	Tibia lead was consistently associated with lower test scores in all 7 cognitive domains Blood lead was not associated with any cognitive domain
Occupational lead exposure									
Lindgren et al. 1996	467	XS	100% 43.4 (11)	White 100	Canadian lead smelter (Canada Lead Study)	370 currently employed 97 previously employed	Blood: 36 IBL^a^: mean across groups, 268.6–1,227.7 TWA^b^: mean across groups, 26.1–52.8	Age, education, language, depressive scale score, head injury, neurological disorder, alcohol use Battery of tests— 8 domains	Lack of association between neuropsychologic performance and blood lead or TWA IBL related to visuomotor skills, psychomotor speed and dexterity, motor speed, and verbal memory performance
Bleecker et al. 1997	80	XS	100 44.1 (8.4)	White 100	Canadian lead smelter (Canada Lead Study)	Current 4–26 years of exposure	Blood: 26.4 (7.1) IBL^a^: 903.1 (305.9) TWA^b^: 42.3 (8.4) Tibia: 41.0 (24.4)	Age, education Battery of tests— 5 domains	Significant amount of variance in verbal memory performance accounted for only by measures of blood lead and TWA Visuomotor ability had significant variance accounted for by measures of TWA, IBL and tibia lead
Chia et al. 1997	50 (E) 97 (NE)	XS	100 (E) 35.7 (10.6) (NE) 33.9 (3.7)	100% Asian (48 Chinese) (E) 100 Asian (43 Chinese) (NE)	Lead battery manufacturing factory (E) Vehicle maintenance workshop (NE)	Current	Blood: (E) 37.1 (range, 13.2–64.6) (NE) 6.14 (range, 2.4–12.4) CumPb^c^: (E) 175.9 (range, 10.0–1146.2)	Age, education, smoking history, ethnic groups, drinking habits Battery of tests— 5 domains	E and NE groups significantly different in tests involving motor dexterity, and visuomotor and psychomotor speed Cumulative blood associated with Digit Symbol and Trail Making Part A scores Cumulative blood lead a stronger predictor of neurobehavioral effects than concurrent blood lead levels
Osterberg et al. 1997	38 (E) 19 (NE)	XS	100 (E) median: 41.5	NR	Secondary lead smelter— inorganic lead (E) Nearby mechancal manufacturing plant (NE)	Current 2–35 years of exposure	Current blood lead^d^: &(E) median, 1.8 (range, 0.9–2.4) (NE) median, 0.18 (range, 0.07–0.34) Peak blood lead^d^: (E) median, 3.0 (range, 2.2–4.3) CBLI^e^: (E) median, 233 (range, 74–948) Finger bone: (E) median, 32 (range, 17–101) (NE) median, 4 (range, –19 to 18)	Matched on age, education, job level Battery of tests—5 domains	Neither blood (current or peak) lead nor finger bone lead levels were associated with any neurobehavioral measures
Hanninen et al. 1998	54	XS	79.6 Low blood lead 41.7 (9.3) High blood lead 46.6 (6.2)	NR	Helsinki lead acid battery factories	Past 12.3, 20.5 years of exposure (means across groups)	Low blood lead^d^: TWA^f^: 1.4 (0.3) Peak blood lead^d^: 1.9 (0.4) IBL^g^: 15.7 (9.5) Calcaneus: 78.6 (62.4) mg/kg Tibia: 19.8 (13.7) mg/kg High blood lead^d^ TWA^f^ : 1.9 (0.4) Peak blood lead^d^: 3.3 (0.7) IBL^g^: 39.2 (18.5) Calcaneus: 100.4 (43.1) mg/kg Tibia: 35.3 (16.6) mg/kg	Age, sex, education Battery of tests— 6 domains	None of the bone lead measures were significantly associated with any test scores The low blood lead group showed associations between historical blood lead measures and visuospatial, visuomotor, attention and verbal comprehension performance The high blood lead group had worse performance than the low blood lead group on tests of attention (Digit Symbol), visual memory (memory for design), and visuoperception (embedded figures)
Stewart et al. 1999	543	XS	100 38% were ≥ 60 years of age (range, 40–70)	White 92.8	Eastern U.S. tetraethyl and tetramethyl lead manufacturing facility (U.S. Organolead Study)	Past Mean of 17.8 years since last exposure at time tibia lead obtained	Tibia: 14.4 (9.3) Peak tibia: 23.7 (17.4)	Age, race, education, testing, lead measures, years since last exposure, depressive score, tobacco, alcohol consumption, visit number Battery of tests— 8 domains	Peak tibia lead strongest and most consistent predictor of test scores: manual dexterity, executive ability, verbal intelligence and memory Current tibia lead also associated with same domains except verbal memory On average, an increase in 22 μg/g peak tibia lead was equivalent to an increase in 5 years of age
Lucchini et al. 2000	66 (E) 86 (NE)	XS	100 (E) 40.1 (8.7) (NE) 42.6 (8.8)	NR	Lead accumulators and bullet manufacturers and 2 lead smelters in Northern Italy (E) Hospital (NE)	Current 1–33 years of exposure	Blood: 27.5 (11.0) (E) 8.1 (4.5) (NE) IBL^a^: 409.8 (360.8) (E) TWA^b^: 31.7 (14.1) (E)	Age, education, alcohol, smoking, coffee intake Neurological symptoms and a battery of 4 neurobehavioral tests	Neurologic symptoms (neuropsychologic, sensory motor) more frequent, and decreased sensitivity to visual contrast sensitivity test in exposed workers. These associations are with current blood lead and not cumulative lead measures (on a E vs. NE comparison, but not individual level) No differences between groups on neurobehavioral tests Significant differences between low and high IBL groups on neuropsychologic scores
Schwartz et al. 2000	535 (E) F/U = 99.8% with 1 + visit 118 (NE) F/U = 91.6% with 1 + visit	L	100 55.6 (7.4) at first visit (E) 58.6 (7.0) (NE)	White 93.1	Eastern U.S. tetraethyl and tetramethyl lead manufacturing facility (E) Community-based random sampling from residential areas of former lead workers (NE) (U.S. Organolead Study)	Past Mean of 16 years since exposure at last baseline	Blood: 4.26 (2.6) (E) Tibia: 14.4 (9.3) (E) Peak tibia: 22.6 (16.5) (E)	Frequency matched on age, education and race Battery of tests— 8 domains	Exposed workers showed greater annual declines than controls in verbal memory, visuoconstruction domains Peak tibia lead, but not blood lead, consistently predicted declines in test scores: symbol digit, verbal and visual memory, motor speed, and executive ability On average, an increase of 15.7 μg/g peak tibia lead was equivalent to annual test decline to ≥ 5 years of age at baseline
Schwartz et al. 2001	803 (E) 135 (NE)	XS	(E) 79.6 40.4 (10.1) (NE) 91.9 34.5 (9.1)	Asian 100	Battery, lead oxide or car radiator manufacturing and secondary lead smelters (E) Air conditioner manufacturing or university (NE) (Korea Lead Study)	Current (8 retired)	Blood: 32 (15) (E) 5.3 (1.8) (NE) Tibia: 37.1 (40.3) (E) 5.8 (7.0) (NE)	Age, sex, education, each lead measure, height, BMI Battery of tests— 9 domains	Blood lead was a better predictor of tests of executive abilities, manual dexterity, and peripheral motor strength than tibia or DMSA-chelatable lead On average, an increase of 5 μg/dL blood lead was equivalent to an increase of 1.05 years in age
Barth et al. 2002	47 (E) 53 (NE)	XS	100 39.5 (9.7) (E) 39.3 (8.4) (NE)	NR	Storage-battery plant (E) Steel production plant (NE) (Austria Lead Study)	Current 0.1–36.1 years of exposure (E)	Blood lead: 30.8 (11.2) (E) 4.32 (2.0) NE) IBL^h^: 4,613.5 (4,187.6) (E)	Age, years of education, verbal intelligence, number of alcoholic drinks per week/grams of alcohol per week Battery of tests— 5 domains	Current blood lead levels, but not cumulative blood lead levels, were correlated with executive functions and visuospatial abilities Executive functioning and visuospatial abilities differed significantly between exposed and control groups
Bleecker et al. 2005	254	XS	100 41 (9.4)	White 100	Canadian lead smelter (Canada Lead Study)	Past	Blood: 27.7 (8.8) IBL^a^: 728.2 (434.4) TWA^b^: 39.0 (12.3)	Age, educational achievement Verbal learning and memory	Significant amount of variance in recognition and delayed recall accounted for only by measures of IBL and TWA The “generalized memory impairment group” had the highest TWA and IBL compared with the “no impairment” and “retrieval difficulties” groups
Schwartz et al. 2005	576 with all visits F/U: all 3 visits, 2 visits, 1 visit = 72%, 16%, 12%	L	76 41.4 (9.5) at visit 1	Asian 100	Battery, lead oxide or car radiator manufacturing and secondary lead smelters (Korea Lead Study)	Current Mean job duration: 8.5 (6.3) (71, 97, 150 no longer working in lead industry at visits 1,2,3)	Blood: 31.4 (14.2) Tibia: 38.4 (43)	Age, education, sex, height, BMI Battery of tests—9 domains	Blood lead cross-sectionally was associated with lower executive ability and manual dexterity scores Change in blood lead was associated with longitudinal declines in few tests Tibia lead was associated with longitudinal declines in manual dexterity, executive abilities, neuropsychiatric and peripheral sensory function
Winker et al. 2005	48 (E) 48 (NE)	XS	100 39.6 (8.8) (E) 39.9 (8.8) (NE)	NR	Storage-battery plant (E) Steel production plant (NE) (Austria Lead Study)	Past Mean of 5.2 years since last exposure	Blood lead: 5.4 (2.7) (E) 4.7 (2.5) (NE) IBL^h^: 4,153.3 (3,690.3) (E)	Age, years of education, verbal intelligence, number of alcoholic drinks per week/grams of alcohol per week Battery of tests—5 domains	Blood lead was correlated with visuospatial abilities, attention, visual scanning, and visuomotor speed. IBL was correlated only with choice reaction No differences between groups on neurobehavioral tests, and no differences between groups stratified by high IBL (> 4,500) vs. low IBL (< 4,500)
Dorsey et al. 2006	652	XS	77 43.4 (9.6)	Asian 100	Battery, lead oxide or car radiator manufacturing and secondary lead smelters (Korea Lead Study)	Current Mean job duration: 10 (6.5)	Blood: 30.9 (16.7) Tibia: 33.5 (43.4) Patella: 75.1 (101.1)	Series of four models adjusting for age, sex, education, job duration, height, BMI Battery of tests—14 neurobehavioral, 4 peripheral nervous system measures, and psychiatric symptoms	Ranked blood lead was associated with executive ability, manual dexterity, and PNS sensory function Ranked tibia lead was similar to blood lead but also associated with psychomotor speed Ranked patella lead was associated with executive ability, manual dexterity, depressive symptoms, and PNS sensory function. Adjustment for blood lead attenuated these associations
Winker et al. 2006	47 (E) 48 (formerly E)	XS	100 39.5 (9.7) (E) 39.6 (8.8) (Formerly E)	NR	Storage-battery plant (E) Storage-battery plant, police officers (Formerly E) (Austria Lead Study)	Current 11.7 (9.0) mean years of exposure duration Past 5.26 (3.5) mean years since last exposure	Blood lead: 30.8 (11.2) (E) 5.4 (2.7) (Formerly E) IBL^h^: 4,613.5 (4,187.6) (E) 4,153.3 (3,690.3) (Formerly E)	Age Battery of tests—5 domains	Visuospatial abilities and executive functioning performance decreased linearly from workers with short exposure duration and long absence from exposure, to the worst performing group with long exposure and short/no absence from exposure

Abbreviations: *APOE*, apolipoprotein E; BMI, body mass index; CI, confidence interval; E, exposed; F/U, follow-up rate; IQR, Interquartile range; L, longitudinal; MMSE, Mini-Mental State Examination; NB, neurobehavioral; NE, nonexposed; Pb, lead; PNS, peripheral nervous system; OR, odds ratio; R, reference; XS, cross-sectional.

Blood lead units: μg/dL; tibia/patella lead units: μg/g (unless noted otherwise). IBL: integrated blood lead calculated from blood measures during a time period, a measure of cumulative dose:

a μg-years/dL;

g μmol-years/l;

h μg-months/dL. TWA: time weighted average calculated by dividing IBL by number of years exposed, a measure of average intensity of lead exposure:

b μg/dL;

f μmol/L.

c CumPb: Area under the curve of blood lead levels over time: μg-years/dL.

d Current and peak blood lead measured in units μmol/L.

e CBLI: cumulative blood lead index: product of blood lead and employment time: μmol-months/L.

## References

[b1-ehp0115-000483] Agency for Toxic Substances and Disease Registry 1999. Toxicological Profile for Lead. Update, 1999. Atlanta: Agency for Toxic Substances and Disease Registry.

[b2-ehp0115-000483] American Psychological Association 2006. PsychINFO Database. Available: http://www.apa.org/psycinfo/ [accessed 24 August 2006].

[b3-ehp0115-000483] Annest JL, Pirkle JL, Makuc D, Neese JW, Bayse DD, Kovar MG (1983). Chronological trend in blood lead levels between 1976 and 1980. N Engl J Med.

[b4-ehp0115-000483] Arnett PA, Higginson CI, Voss WD, Bender WI, Wurst JM, Tippin JM (1999). Depression in multiple sclerosis: relationship to working memory capacity. Neuropsychology.

[b5-ehp0115-000483] Baker EL, Feldman RG, White RF, Harley JP (1983). The role of occupational lead exposure in the genesis of psychiatric and behavioral disturbances. Acta Psychiatr Scand Suppl.

[b6-ehp0115-000483] Balbus-Kornfeld JM, Stewart W, Bolla KI, Schwartz BS (1995). Cumulative exposure to inorganic lead and neurobehavioural test performance in adults: an epidemiological review. Occup Environ Med.

[b7-ehp0115-000483] Barth A, Schaffer AW, Osterode W, Winker R, Konnaris C, Valic E (2002). Reduced cognitive abilities in lead-exposed men. Int Arch Occup Environ Health.

[b8-ehp0115-000483] Birkenhager WH, Staessen JA (2006). Progress in cardiovascular diseases: cognitive function in essential hypertension. Prog Cardiovasc Dis.

[b9-ehp0115-000483] Bleecker ML, Ford DP, Lindgren KN, Hoese VM, Walsh KS, Vaughan CG (2005). Differential effects of lead exposure on components of verbal memory. Occup Environ Med.

[b10-ehp0115-000483] Bleecker ML, Lindgren KN, Ford DP (1997). Differential contribution of current and cumulative indices of lead dose to neuropsychological performance by age. Neurology.

[b11-ehp0115-000483] Chia SE, Chia HP, Ong CN, Jeyaratnam J (1997). Cumulative blood lead levels and neurobehavioral test performance. Neurotoxicology.

[b12-ehp0115-000483] Corder EH, Saunders AM, Strittmatter WJ, Schmechel DE, Gaskell PC, Small GW (1993). Gene dose of apolipoprotein E type 4 allele and the risk of Alzheimer’s disease in late onset families. Science.

[b13-ehp0115-000483] Cory-Slechta DA (1995). Relationships between lead-induced learning impairments and changes in dopaminergic, cholinergic, and glutamatergic neurotransmitter system functions. Annu Rev Pharmacol Toxicol.

[b14-ehp0115-000483] Derogatis LR, Melisaratos N (1983). The Brief Symptom Inventory: an introductory report. Psychol Med.

[b15-ehp0115-000483] Dufouil C, Alperovitch A, Ducros V, Tzourio C (2003). Homocysteine, white matter hyperintensities, and cognition in healthy elderly people. Ann Neurol.

[b16-ehp0115-000483] Eichenbaum H (2001). The hippocampus and declarative memory: cognitive mechanisms and neural codes. Behav Brain Res.

[b17-ehp0115-000483] Ferguson C, Kern M, Audesirk G (2000). Nanomolar concentrations of inorganic lead increase Ca^2+^ efflux and decrease intracellular free Ca^2+^ ion concentrations in cultured rat hippocampal neurons by a calmodulin-dependent mechanism. Neurotoxicology.

[b18-ehp0115-000483] Finkelstein Y, Markowitz ME, Rosen JF (1998). Low-level lead-induced neurotoxicity in children: an update on central nervous system effects. Brain Res Rev.

[b19-ehp0115-000483] Folstein MF, Folstein SE, McHugh PR (1975). “Minimental state”. A practical method for grading the cognitive state of patients for the clinician”. J Psychiatr Res.

[b20-ehp0115-000483] Glenn BS, Bandeen-Roche K, Lee BK, Weaver VM, Todd AC, Schwartz BS (2006). Changes in systolic blood pressure associated with lead in blood and bone. Epidemiology.

[b21-ehp0115-000483] Glenn BS, Stewart WF, Links JM, Todd AC, Schwartz BS (2003). The longitudinal association of lead with blood pressure. Epidemiology.

[b22-ehp0115-000483] Goodman M, LaVerda N, Clarke C, Foster ED, Iannuzzi J, Mandel J (2002). Neurobehavioural testing in workers occupationally exposed to lead: systematic review and meta-analysis of publications. Occup Environ Med.

[b23-ehp0115-000483] Goodman M, LaVerda N, Mandel J (2001). Commentary on “A meta-analysis for neurobehavioural results due to occupational lead exposure with blood lead concentrations <70 microg/100 ml” by M. Meyer-Baron and A. Seeber. Arch Toxicol.

[b24-ehp0115-000483] Guallar E, Silbergeld EK, Navas-Acien A, Malhotra S, Astor BC, Sharrett AR (2006). Confounding of the relation between homocysteine and peripheral arterial disease by lead, cadmium, and renal function. Am J Epidemiol.

[b25-ehp0115-000483] Hanninen H, Aitio A, Kovala T, Luukkonen R, Matikainen E, Mannelin T (1998). Occupational exposure to lead and neuropsychological dysfunction. Occup Environ Med.

[b26-ehp0115-000483] Hayden KM, Zandi PP, Lyketsos CG, Khachaturian AS, Bastian LA, Charoonruk G (2006). Vascular risk factors for incident Alzheimer disease and vascular dementia: the Cache County study. Alzheimer Dis Assoc Disord.

[b27-ehp0115-000483] Hogervorst E, Ribeiro HM, Molyneux A, Budge M, Smith AD (2002). Plasma homocysteine levels, cerebrovascular risk factors, and cerebral white matter changes (leukoaraiosis) in patients with Alzheimer disease. Arch Neurol.

[b28-ehp0115-000483] Hoppin JA, Aro A, Hu H, Ryan PB (2000). Measurement variability associated with KXRF bone lead measurement in young adults. Environ Health Perspect.

[b29-ehp0115-000483] Hoppin JA, Aro AC, Williams PL, Hu H, Ryan PB (1995). Validation of K-XRF bone lead measurement in young adults. Environ Health Perspect.

[b30-ehp0115-000483] Hu H, Shih R, Rothenberg S, Schwartz BS (2007). The epidemiology of lead toxicity in adults: measuring dose and consideration of other methodologic issues. Environ Health Perspect.

[b31-ehp0115-000483] Jorm AF (2000). Is depression a risk factor for dementia or cognitive decline? A review. Gerontology.

[b32-ehp0115-000483] Kosnett MJ, Wedeen RP, Rothenberg SJ, Hipkins KL, Materna BL, Schwartz BS (2007). Recommendations for medical management of adult lead exposure. Environ Health Perspect.

[b33-ehp0115-000483] Lindgren KN, Masten VL, Ford DP, Bleecker ML (1996). Relation of cumulative exposure to inorganic lead and neuropsychological test performance. Occup Environ Med.

[b34-ehp0115-000483] Links JM, Schwartz BS, Simon D, Bandeen-Roche K, Stewart WF (2001). Characterization of toxicokinetics and toxicodynamics with linear systems theory: application to lead-associated cognitive decline. Environ Health Perspect.

[b35-ehp0115-000483] Lucchini R, Albini E, Cortesi I, Placidi D, Bergamaschi E, Traversa F (2000). Assessment of neurobehavioral performance as a function of current and cumulative occupational lead exposure. Neurotoxicology.

[b36-ehp0115-000483] Maizlish NA, Parra G, Feo O (1995). Neurobehavioral evaluation of Venezuelan workers exposed to inorganic lead. Occup Environ Med.

[b37-ehp0115-000483] Martin D, Glass TA, Bandeen-Roche K, Todd AC, Shi W, Schwartz BS (2006). Association of blood lead and tibia lead with blood pressure and hypertension in a community sample of older adults. Am J Epidemiol.

[b38-ehp0115-000483] Mayeux R, Ottman R, Maestre G, Ngai C, Tang MX, Ginsberg H (1995). Synergistic effects of traumatic head injury and apolipoproteinepsilon 4 in patients with Alzheimer’s disease. Neurology.

[b39-ehp0115-000483] McCaddon A, Hudson P, Hill D, Barber J, Lloyd A, Davies G (2003). Alzheimer’s disease and total plasma aminothiols. Biol Psychiatry.

[b40-ehp0115-000483] McCaddon A, Kelly CL (1992). Alzheimer’s disease: a ‘cobalaminergic’ hypothesis. Med Hypotheses.

[b41-ehp0115-000483] McNair DM Lorr M Droppelman LF 1971. Manual for the Profile of Mood States. San Diego, CA:Educational and Industrial Testing Service.

[b42-ehp0115-000483] Meyer MR, Tschanz JT, Norton MC, Welsh-Bohmer KA, Steffens DC, Wyse BW (1998). APOE genotype predicts when—not whether—one is predisposed to develop Alzheimer disease. Nat Genet.

[b43-ehp0115-000483] Meyer-Baron M, Seeber A (2000). A meta-analysis for neurobehavioural results due to occupational lead exposure with blood lead concentrations <70 microg/100 ml. Arch Toxicol.

[b44-ehp0115-000483] Miyata M, Smith JD (1996). Apolipoprotein E allele-specific antioxidant activity and effects on cytotoxicity by oxidative insults and beta-amyloid peptides. Nat Genet.

[b45-ehp0115-000483] Moffat SD, Szekely CA, Zonderman AB, Kabani NJ, Resnick SM (2000). Longitudinal change in hippocampal volume as a function of apolipoprotein E genotype. Neurology.

[b46-ehp0115-000483] Naismith SL, Hickie IB, Turner K, Little CL, Winter V, Ward PB (2003). Neuropsychological performance in patients with depression is associated with clinical, etiological and genetic risk factors. J Clin Exp Neuropsychol.

[b47-ehp0115-000483] Nash D, Magder L, Lustberg M, Sherwin RW, Rubin RJ, Kaufmann RB (2003). Blood lead, blood pressure, and hypertension in perimenopausal and postmenopausal women. JAMA.

[b48-ehp0115-000483] National Library of Medicine 2006. PubMed Database. Available: http://www.ncbi.nlm.nih.gov/entrez/query.fcgi?DB=pubmed [accessed 24 August 2006].

[b49-ehp0115-000483] Osterberg K, Borjesson J, Gerhardsson L, Schutz A, Skerfving S (1997). A neurobehavioural study of long-term occupational inorganic lead exposure. Sci Total Environ.

[b50-ehp0115-000483] Parnetti L, Bottiglieri T, Lowenthal D (1997). Role of homocysteine in age-related vascular and non-vascular diseases. Aging (Milano).

[b51-ehp0115-000483] Payton M, Riggs KM, Spiro A, Weiss ST, Hu H (1998). Relations of bone and blood lead to cognitive function: the VA Normative Aging Study. Neurotoxicol Teratol.

[b52-ehp0115-000483] Petersen RC, Smith GE, Waring SC, Ivnik RJ, Tangalos EG, Kokmen E (1999). Mild cognitive impairment: clinical characterization and outcome. Arch Neurol.

[b53-ehp0115-000483] Pirkle JL, Kaufmann RB, Brody DJ, Hickman T, Gunter EW, Paschal DC (1998). Exposure of the U.S. population to lead, 1991–1994. Environ Health Perspect.

[b54-ehp0115-000483] Poirier J, Sevigny P (1998). Apolipoprotein E4, cholinergic integrity and the pharmacogenetics of Alzheimer’s disease. J Neural Transm Suppl.

[b55-ehp0115-000483] Radloff LS (1977). The CES-D scale: a self report depression scale for research in the general population. Appl Psychol Measurement.

[b56-ehp0115-000483] Ramesh GT, Manna SK, Aggarwal BB, Jadhav AL (2001). Lead exposure activates nuclear factor kappa B, activator protein-1, c-Jun N-terminal kinase and caspases in the rat brain. Toxicol Lett.

[b57-ehp0115-000483] Rhodes D, Spiro A, Aro A, Hu H (2003). Relationship of bone and blood lead levels to psychiatric symptoms: the normative aging study. J Occup Environ Med.

[b58-ehp0115-000483] Salthouse TA (1996). General and specific speed mediation of adult age differences in memory. J Gerontol B Psychol Sci Soc Sci.

[b59-ehp0115-000483] Salthouse TA (1996). The processing-speed theory of adult age differences in cognition. Psychol Rev.

[b60-ehp0115-000483] Saunders AM, Schmader K, Breitner JC, Benson MD, Brown WT, Goldfarb L (1993). Apolipoprotein E epsilon 4 allele distributions in late-onset Alzheimer’s disease and in other amyloid-forming diseases. Lancet.

[b61-ehp0115-000483] Schafer JH, Glass TA, Bolla KI, Mintz M, Jedlicka AE, Schwartz BS (2005a). Homocysteine and cognitive function in a population-based study of older adults. J Am Geriatr Soc.

[b62-ehp0115-000483] Schafer JH, Glass TA, Bressler J, Todd AC, Schwartz BS (2005b). Blood lead is a predictor of homocysteine levels in a population-based study of older adults. Environ Health Perspect.

[b63-ehp0115-000483] Schwartz BS, Lee BK, Bandeen-Roche K, Stewart W, Bolla K, Links J (2005). Occupational lead exposure and longitudinal decline in neurobehavioral test scores. Epidemiology.

[b64-ehp0115-000483] Schwartz BS, Lee BK, Lee GS, Stewart WF, Lee SS, Hwang KY (2001). Associations of blood lead, dimercaptosuccinic acid-chelatable lead, and tibia lead with neurobehavioral test scores in South Korean lead workers. Am J Epidemiol.

[b65-ehp0115-000483] Schwartz BS, Stewart W, Hu H (2002). Neurobehavioural testing in workers occupationally exposed to lead. Occup Environ Med.

[b66-ehp0115-000483] Schwartz BS, Stewart WF, Bolla KI, Simon PD, Bandeen-Roche K, Gordon PB (2000). Past adult lead exposure is associated with longitudinal decline in cognitive function. Neurology.

[b67-ehp0115-000483] Seeber A, Meyer-Baron M (2003). Neurobehavioural testing in workers occupationally exposed to lead. Occup Environ Med.

[b68-ehp0115-000483] Seeber A, Meyer-Baron M, Schaper M (2002). A summary of two meta-analyses on neurobehavioural effects due to occupational lead exposure. Arch Toxicol.

[b69-ehp0115-000483] Selley ML (2003). Increased concentrations of homocysteine and asymmetric dimethylarginine and decreased concentrations of nitric oxide in the plasma of patients with Alzheimer’s disease. Neurobiol Aging.

[b70-ehp0115-000483] Shih RA, Glass TA, Bandeen-Roche K, Carlson MC, Bolla KI, Todd AC (2006). Environmental lead exposure and cognitive function in community-dwelling older adults. Neurology.

[b71-ehp0115-000483] Skoog I, Gustafson D (2006). Update on hypertension and Alzheimer’s disease. Neurol Res.

[b72-ehp0115-000483] Stewart WF, Schwartz BS, Davatzikos C, Shen D, Liu D, Wu X (2006). Past adult lead exposure is linked to neurodegeneration measured by brain MRI. Neurology.

[b73-ehp0115-000483] Stewart WF, Schwartz BS, Simon D, Bolla KI, Todd AC, Links J (1999). Neurobehavioral function and tibial and chelatable lead levels in 543 former organolead workers. Neurology.

[b74-ehp0115-000483] Stewart WF, Schwartz BS, Simon D, Kelsey K, Todd AC (2002). ApoE genotype, past adult lead exposure, and neurobehavioral function. Environ Health Perspect.

[b75-ehp0115-000483] Stokes L, Letz R, Gerr F, Kolczak M, McNeill FE, Chettle DR (1998). Neurotoxicity in young adults 20 years after childhood exposure to lead: the Bunker Hill experience. Occup Environ Med.

[b76-ehp0115-000483] Teter B, Xu PT, Gilbert JR, Roses AD, Galasko D, Cole GM (1999). Human apolipoprotein E isoform-specific differences in neuronal sprouting in organotypic hippocampal culture. J Neurochem.

[b77-ehp0115-000483] Weingartner H, Cohen RM, Murphy DL, Martello J, Gerdt C (1981). Cognitive processes in depression. Arch Gen Psychiatry.

[b78-ehp0115-000483] Weiss B, Bellinger DC (2006). Social ecology of children’s vulnerability to environmental pollutants. Environ Health Perspect.

[b79-ehp0115-000483] Weisskopf MG, Proctor SP, Wright RO, Schwartz J, Spiro A, Sparrow D (2007). Cumulative lead exposure and cognitive performance among elderly men. Epidemiology.

[b80-ehp0115-000483] Weisskopf MG, Wright RO, Schwartz J, Spiro A, Sparrow D, Aro A (2004). Cumulative lead exposure and prospective change in cognition among elderly men: the VA Normative Aging Study. Am J Epidemiol.

[b81-ehp0115-000483] Widzowski DV, Cory-Slechta DA (1994). Homogeneity of regional brain lead concentrations. Neurotoxicology.

[b82-ehp0115-000483] Wilson RS, Beckett LA, Barnes LL, Schneider JA, Bach J, Evans DA (2002). Individual differences in rates of change in cognitive abilities of older persons. Psychol Aging.

[b83-ehp0115-000483] Wright RO, Tsaih SW, Schwartz J, Spiro A, McDonald K, Weiss ST (2003). Lead exposure biomarkers and mini-mental status exam scores in older men. Epidemiology.

[b84-ehp0115-000483] Yankner BA (1996). Mechanisms of neuronal degeneration in Alzheimer’s disease. Neuron.

[b85-ehp0115-000483] Zawia NH, Crumpton T, Brydie M, Reddy GR, Razmiafshari M (2000). Disruption of the zinc finger domain: a common target that underlies many of the effects of lead. Neurotoxicology.

[b86-ehp0115-000483] Zubenko GS, Stiffler S, Stabler S, Kopp U, Hughes HB, Cohen BM (1994). Association of the apolipoprotein E epsilon 4 allele with clinical subtypes of autopsy-confirmed Alzheimer’s disease. Am J Med Genet.

